# Having few remaining teeth is associated with a low nutrient intake and low serum albumin levels in middle-aged and older Japanese individuals: findings from the NIPPON DATA2010

**DOI:** 10.1186/s12199-018-0752-x

**Published:** 2019-01-05

**Authors:** Mieko Nakamura, Toshiyuki Ojima, Tomomi Nagahata, Imako Kondo, Toshiharu Ninomiya, Katsushi Yoshita, Yusuke Arai, Takayoshi Ohkubo, Keiko Murakami, Nobuo Nishi, Yoshitaka Murakami, Naoyuki Takashima, Nagako Okuda, Aya Kadota, Naoko Miyagawa, Keiko Kondo, Tomonori Okamura, Hirotsugu Ueshima, Akira Okayama, Katsuyuki Miura, for the NIPPON DATA2010 Research Group

**Affiliations:** 1 Department of Community Health and Preventive Medicine, Hamamatsu University School of Medicine, 1-20-1 Handayama, Higashi-ku, Hamamatsu, Shizuoka 431-3192 Japan; 2 Department of Nutrition, School of Health and Nutrition, Tokaigakuen University, Nagoya, Japan; 3 Department of Food and Nutritional Sciences, College of Bioscience and Biotechnology, Chubu University, Kasugai, Japan; 4 Department of Epidemiology and Public Health, Graduate School of Medical Sciences, Kyushu University, Fukuoka, Japan; 5 Department of Food Science and Nutrition, Osaka City University, Osaka, Japan; 6 Department of Nutrition, Chiba Prefectural University of Health Sciences, Chiba, Japan; 7 Department of Hygiene and Public Health, Teikyo 451University School of Medicine, Tokyo, Japan; 8 Tohoku Medical Megabank Organization, Tohoku University, Sendai, Japan; 9 International Center for Nutrition and Information, National Institute of Health and Nutrition, National Institutes of Biomedical Innovation, Health and Nutrition, Tokyo, Japan; 10 Department of Medical Statistics, Toho University School of Medicine, Tokyo, Japan; 11 Department of Public Health, Shiga University of Medical Science, Otsu, Japan; 12 Department of Health and Nutrition, University of Human Arts and Sciences, Saitama, Japan; 13 Center for Epidemiologic Research in Asia, Shiga University of Medical Science, Otsu, Japan; 14 Department of Preventive Medicine and Public Health, School of Medicine, Keio University, Tokyo, Japan; 15 Research Institute of Strategy for Prevention, Tokyo, Japan

**Keywords:** Oral health, Diet, Albumin, Socioeconomic status, Older people

## Abstract

**Background:**

Oral health is thought to be associated with diet quality, and socioeconomic status (SES) affects both oral health and diet. The aim of this study was to investigate the association between the number of teeth and dietary intake as well as nutritional biomarker, considering the subjects’ SES.

**Methods:**

We conducted a cross-sectional analysis of data from 2049 individuals aged ≥ 50 years from the National Integrated Project for Prospective Observation of Non-communicable Disease and its Trends in the Aged 2010. The number of remaining teeth was categorized into age-specific quartiles (Q1 to Q4). We assessed the adjusted means and 95% confidence intervals for dietary variables by the number of teeth using analysis of covariance. Stratified analyses by SES were also conducted.

**Results:**

The intake of grain products was 31 g higher, and those of vegetables and meat were 30 g and 8 g lower, respectively, in Q1 (fewer teeth) than in Q4 (more teeth). Carbohydrate intake was higher whereas protein, minerals (potassium, magnesium, and zinc), vitamins (vitamins A, E, B_1_, B_6_, β-carotene, and folic acid), and dietary fiber intakes were lower among individuals with fewer teeth. Adjusted mean serum albumin levels were low in Q1. The associations between the number of teeth and dietary intake were more evident in individuals with a low SES.

**Conclusions:**

Having few remaining teeth was associated with a low nutrient intake and low serum albumin levels in middle-aged and older Japanese adults, and these associations were more evident in individuals with low SES.

**Electronic supplementary material:**

The online version of this article (10.1186/s12199-018-0752-x) contains supplementary material, which is available to authorized users.

## Introduction

Diet is closely related to health [1]. Oral health is thought to be associated with diet quality, especially in older people [2]. Previous reports suggest that there is an association between poor oral health and a low intake of certain types of foods [3–12]. A low intake of such foods can result in a reduced intake of some nutrients [3–7, 9, 10, 12–15] and low levels of nutritional biomarkers such as serum albumin [16, 17]. Maintaining an adequate serum albumin concentration is especially important for older people, because a low serum albumin concentration has been reported to be a predictive factor for frailty [18] and lower survival [19]. However, the association between poor oral health and nutritional biomarker levels has not been well documented [4, 16, 17, 20].

Socioeconomic status (SES) is also associated with oral health [21] and dietary intake [22–24]. Individuals with a low SES tend to have a poorer diet quality, including a low intake of vitamins and minerals [22]. The reason for this finding might be that foods with a cheaper cost per energy, which are frequently chosen by individuals with a low SES, tend to be energy-dense but nutrient-poor. Therefore, it is important to consider SES in studies investigating the association between oral health and dietary intake.

However, to date, only a few studies have reported the association between oral health and dietary intake considering the subjects’ SES [8]. Accordingly, this study aimed to evaluate the associations between oral health, as measured by the number of remaining teeth, and dietary intake, and the associations after stratifying by SES, in a representative population of middle-aged and older Japanese adults.

## Materials and methods

### Study design

The National Integrated Project for Prospective Observation of Non-communicable Disease and its Trends in the Aged 2010 (NIPPON DATA2010), which was initiated in 2010, is a prospective cohort study that investigates cardiovascular disease in Japan. The baseline survey of this study was conducted using the National Health and Nutrition Survey (NHNS) in November 2010 and the Comprehensive Survey of Living Conditions (CSLC) in June 2010, both of which were implemented by the Ministry of Health, Labour and Welfare. The details of the NHNS 2010 [25, 26], CSLC 2010 [27, 28], and the NIPPON DATA2010 [29] have been described elsewhere.

### Participants

Briefly, 8815 individuals aged ≥ 1 year residing in 300 randomly selected districts throughout Japan participated in the NHNS 2010. Of 7229 individuals aged ≥ 20 years, 3873 underwent blood examinations in the NHNS 2010. Of these, 2898 agreed to participate in the NIPPON DATA2010. The data of 7 persons who could not be merged with the NHNS 2010 were excluded from the dataset; thus, the data of 2891 participants were included. We analyzed the data of 2049 participants aged ≥ 50 years, because the rate of tooth loss is very low in the younger generation, and complete tooth loss was only seen in some individuals in their 50s and in those who were older in the NIPPON DATA2010.

### Number of teeth, SES, and other variables

Self-administered questionnaires were used to obtain information on the number of teeth, smoking status, use of antidiabetic medications, annual household income, and the number of family members (NHNS 2010); equivalent household expenditure per month (CSLC 2010); and educational attainment (NIPPON DATA2010). All information was carefully reviewed by trained interviewers. Blood samples were taken after 4 h of fasting in the NHNS 2010. Tooth counts were obtained using the question: “How many natural teeth do you have? The natural teeth include dental crowns, but do not include wisdom teeth (third molars), dentures, bridges, and implants.” with the answer: “I have (blank space) natural teeth.” The self-reported number of remaining teeth was divided into age-specific quartiles to control for the cofounding of age, because the number of teeth is highly age-dependent.

SES was divided into high and low, using annual household income, equivalent household expenditure per month, and educational attainment. Annual household income in the previous year was obtained as the categorical variable and classified into low (< 2 million Japanese yen [JPY]) and high (≥ 2 million JPY). Participants who answered “do not know” were excluded from the analysis. In the analysis of the annual household income, the square root of the number of household members was adjusted for. Equivalent household expenditure per month was obtained by using the household expenses in May 2010 divided by the square root of the number of household members, and dichotomized using the median (low, < 133 thousand JPY; high, ≥ 133 thousand JPY). Educational attainment was categorized into low (up to high school) and high (college or higher).

### Dietary assessment

Dietary intake was assessed with the “1-day household-based food weighing with approximated proportions method” in the NHNS 2010 [25, 30]. This method was developed to monitor the nationwide nutritional status of individual participants in Japan using a household-based dietary survey [25, 30]. Nutrient intake was estimated based on the food composition table in Japan [31]. Grain products were divided into rice, bread, noodles, and others. The proportion of total energy (% energy) was used for energy-yielding nutrients, and the energy-adjusted intake by density method (/1000 kcal) was used for other nutrients and foods.

### Statistical analysis

We examined the differences in participant characteristics by the number of teeth by analysis of variance for continuous variables and the chi-squared test for categorical variables. Then, adjusted means and 95% confidence intervals for the intakes of food groups and nutrients as well as serum albumin and hemoglobin levels by the number of teeth were obtained by analysis of covariance. We adjusted the model for age, sex, smoking status, and the use of antidiabetic medications, because preliminary stratified analyses by sex did not show any significant differences in the association between dietary variables and the number of teeth. On the other hand, sub-group analyses by sex were performed for serum albumin and hemoglobin concentrations, because the association between these variables and the number of teeth was somewhat different by sex.

Sub-group analyses by SES were performed for the intakes of grain products, vegetables, and meat, as well as serum albumin concentration. The interaction between the number of teeth and SES was examined by inserting the number of teeth*SES as an interaction term.

Pairwise deletion was used for missing data. All statistical analyses were performed with IBM SPSS Statistics 22 (IBM, New York, USA). All tests of significance were 2-tailed, with *P* < 0.05 considered significant.

## Results

### Characteristics of the subjects

We determined the age-specific quartiles for the number of teeth (Q1 to Q4). Characteristics of the subjects by the number of teeth are shown in Table 1. The age differences among the four groups were statistically significant but very small. The proportion of low SES (household income, equivalent household expenditure, and educational attainment) was higher in participants with fewer teeth than in those with more teeth.

**Table 1:** Characteristics of the subjects by the number of teeth (brackets contain percent)

	Number of teeth				*p* value^1^
	Q1	Q2	Q3	Q4	
Number of teeth, range					
50–59	0–19	20–25	26–27	28	
60–69	0–14	15–21	22–26	27–28	
70–79	0–6	7–17	18–24	25–28	
80-	0	1–7	8–19	20–28	
*n*	493	485	529	542	
Sex					
Men	242 (49.1)	204 (42.1)	223 (42.2)	239 (44.1)	0.09
Women	251 (50.9)	281 (57.9)	306 (57.8)	303 (55.9)	
Age, mean, SD	68.5, 9.4	66.5, 9.3	66.9, 8.5	66.0, 9.2	< 0.01
Age group					
50–59	97 (19.7)	131 (27.0)	94 (17.8)	143 (26.4)	< 0.01
60–69	190 (38.5)	161 (33.2)	240 (45.4)	195 (36.0)	
70–79	145 (29.4)	153 (31.5)	146 (27.6)	152 (28.0)	
80-	61 (12.4)	40 (8.2)	49 (9.3)	52 (9.6)	
Smoking					
Former smoker	110 (22.4)	92 (19.0)	114 (21.6)	110 (20.3)	< 0.01
Current smoker	93 (18.9)	64 (13.2)	52 (9.8)	46 (8.5)	
Use of antidiabetic medications	65 (13.2)	43 (8.9)	36 (6.8)	42 (7.8)	< 0.01
Household income^2^					
Low	148 (33.9)	117 (26.5)	108 (22.5)	94 (18.9)	< 0.01
High	289 (66.1)	324 (73.5)	372 (77.5)	404 (81.1)	
Equivalent household expenditure^3^					
Low	273 (61.1)	227 (51.6)	209 (42.1)	233 (45.3)	< 0.01
High	174 (38.9)	213 (48.4)	287 (57.9)	281 (54.7)	
Educational attainment^4^					
Low	414 (84.5)	381 (78.9)	408 (77.1)	385 (71.0)	< 0.01
High	76 (15.5)	102 (21.1)	121 (22.9)	157 (29.0)	

^1^Analysis of variance for continuous variable and chi-square test for categorical variable

^2^Low, < 2 million JPY; high, ≥ 2 million JPY

^3^Low, < 133 thousand JPY; high, ≥ 133 thousand JPY

^4^Low, up to high school; high, college or more

### Food group intake by the number of teeth

The adjusted means for food group intake by the number of teeth are shown in Table 2 (crude intake) and Additional file 1: Table S1 (energy-adjusted intake). The number of teeth was inversely associated with the intake of grain products. The crude intake of grain products was higher in individuals with fewer teeth (31 g higher for Q1 than Q4). This inverse association was significant for rice but not for bread and noodles. The number of teeth was positively associated with meat and vegetable intake. The intakes of vegetables and meat were 30 g and 8 g lower, respectively, in participants with fewer teeth (Q1) than in those with more teeth (Q4).

**Table 2:** Adjusted means of food group intake by the number of teeth (brackets contain 95% confidence intervals)

	Number of teeth				Trend *p* value
	Q1	Q2	Q3	Q4	
Grain products, g	460 (447–474)	446 (433–459)	427 (414–440)	429 (417–442)	< 0.01
Rice, g	342 (328–357)	338 (324–353)	316 (302–330)	326 (312–340)	0.03
Bread, g	39 (35–43)	36 (32–40)	38 (34–42)	34 (30–38)	0.18
Noodles, g	62 (53–71)	59 (51–68)	58 (50–67)	52 (44–60)	0.10
Potatoes, g	61 (55–68)	63 (57–70)	60 (54–66)	64 (58–70)	0.72
Soy and soy products, g	74 (66–81)	71 (64–78)	75 (68–81)	73 (66–79)	0.95
Vegetables, g	301 (285–318)	328 (312–344)	340 (324–355)	331 (316–347)	< 0.01
Fruits, g	136 (124–148)	143 (131–155)	151 (139–162)	143 (132–155)	0.30
Fishes and Shellfishes, g	91 (84–97)	91 (84–97)	98 (92–105)	91 (85–98)	0.54
Meats, g	60 (55–65)	62 (57–67)	66 (61–71)	68 (63–73)	0.01
Eggs, g	34 (31–37)	37 (34–40)	38 (35–41)	35 (32–37)	0.75
Milks, g	95 (84–106)	113 (102–124)	117 (106–128)	109 (99–120)	0.07
Beverages, g	719 (676–762)	730 (688–773)	760 (719–801)	747 (707–788)	0.23

Age, sex, smoking status, and the use of antidiabetic medications were adjusted

### Nutrient intake by the number of teeth

The adjusted means for the intake of nutrients by the number of teeth are shown in Table 3 (crude intake) and Additional file 1: Table S2 (energy-adjusted intake). The number of teeth was inversely associated with the energy-adjusted intake of carbohydrates. In contrast, it was positively associated with the crude and energy-adjusted intakes of protein (total and animal), minerals (potassium, magnesium, and zinc), some vitamins (vitamins A, E, B_1_, B_6_, β-carotene, and folic acid), and dietary fiber. However, the number of teeth was not associated with the intakes of total energy, fat except mono-unsaturated fatty acids, and sodium in both crude and energy-adjusted (other than total energy) intake. Sex stratification did not substantively change these results (data not shown).

**Table 3:** Adjusted means of nutrient intake by the number of teeth (brackets contain 95% confidence intervals)

	Number of teeth				Trend *p* value
	Q1	Q2	Q3	Q4	
Total energy, kcal	1908 (1866–1949)	1915 (1873–1956)	1948 (1909–1988)	1914 (1874–1953)	0.61
Carbohydrate, g	276.8 (270.3–283.3)	274.3 (267.9–280.8)	272.5 (266.3–278.7)	271.3 (265.1–277.4)	0.20
Dietary fiber, g	16.3 (15.7–17.0)	17.0 (16.4–17.6)	17.5 (16.9–18.2)	17.3 (16.7–17.9)	0.01
Protein, g	70.0 (68.1–71.8)	70.9 (69.0–72.8)	74.0 (72.3–75.8)	72.5 (70.7–74.3)	0.01
Animal protein, g	34.8 (33.3–36.3)	36.2 (34.7–37.7)	39.4 (37.9–40.8)	38.0 (36.5–39.4)	< 0.01
Vegetable protein, g	35.1 (34.2–36.1)	34.7 (33.7–35.6)	34.7 (33.8–35.6)	34.5 (33.6–35.4)	0.39
Fat, g	49.7 (47.9–51.5)	51.1 (49.3–52.9)	53.2 (51.4–54.9)	51.3 (49.6–53.0)	0.11
SFA, g	13.0 (12.5–13.6)	13.5 (12.9–14.0)	13.9 (13.3–14.4)	13.4 (12.8–13.9)	0.28
MUFA, g	16.3 (15.6–17.0)	17.0 (16.3–17.7)	17.6 (17.0–18.3)	17.2 (16.6–17.9)	0.04
PUFA, g	11.7 (11.2–12.2)	11.9 (11.4–12.4)	12.0 (11.5–12.4)	11.9 (11.4–12.3)	0.66
Sodium, mg	4422 (4269–4574)	4451 (4300–4603)	4584 (4438–4729)	4386 (4242–4530)	0.98
Potassium, mg	2460 (2377–2543)	2602 (2519–2685)	2685 (2605–2764)	2639 (2560–2718)	< 0.01
Calcium, mg	565 (539–590)	582 (557–608)	612 (588–637)	592 (568–616)	0.06
Magnesium, mg	266 (257–274)	276 (267–284)	284 (276–293)	279 (271–287)	0.01
Iron, mg	8.5 (8.3–8.8)	8.7 (8.4–9.0)	9.1 (8.8–9.4)	8.9 (8.6–9.2)	0.04
Zinc, mg	7.9 (7.7–8.1)	8.1 (7.8–8.3)	8.3 (8.1–8.5)	8.2 (8.0–8.5)	< 0.01
Vitamin A, μgRE	528 (478–579)	572 (522–623)	629 (580–677)	627 (579–675)	< 0.01
β-carotene, μg	4306 (3976–4636)	4764 (4436–5093)	5072 (4757–5387)	5048 (4735–5360)	< 0.01
Vitamin D, μg	9.1 (8.3–9.9)	9.1 (8.3–10.0)	9.7 (8.9–10.5)	9.4 (8.6–10.2)	0.46
Vitamin E, mg	8.7 (7.0–10.5)	8.6 (6.9–10.4)	11.2 (9.5–12.9)	11.4 (9.7–13.1)	< 0.01
Vitamin K, μg	266 (248–284)	272 (254–290)	282 (265–299)	287 (270–304)	0.07
Vitamin B_1_, mg	1.73 (0.91–2.55)	2.08 (1.26–2.89)	2.32 (1.54–3.11)	2.92 (2.14–3.70)	0.04
Vitamin B_2_, mg	1.58 (1.11–2.05)	1.58 (1.11–2.05)	1.86 (1.41–2.31)	2.09 (1.65–2.54)	0.08
Vitamin B_6_, mg	1.76 (0.91–2.60)	2.47 (1.63–3.31)	2.79 (1.98–3.59)	3.43 (2.63–4.23)	< 0.01
Vitamin B_12_, μg	6.9 (6.3–7.5)	7.0 (6.4–7.6)	7.3 (6.7–7.9)	7.6 (7.0–8.2)	0.08
Folic acid, μg	327 (313–341)	349 (335–363)	356 (343–369)	360 (347–373)	< 0.01
Vitamin C, mg	133 (120–146)	135 (122–147)	149 (137–162)	146 (134–158)	0.07

Age, sex, smoking status, and the use of antidiabetic medications were adjusted

### Blood biomarker levels by the number of teeth

Few individuals (2.7%) had clinically low serum albumin levels (< 4.0 g/dL). Table 4 shows the association between serum albumin and hemoglobin levels and the number of teeth.

**Table 4:** Adjusted means of blood biomarker levels by the number of teeth (brackets contain 95% confidence intervals)

	Number of teeth				Trend *p* value
	Q1	Q2	Q3	Q4	
Serum albumin, g/dl^1^	4.40 (4.38–4.43)	4.44 (4.42–4.46)	4.44 (4.41–4.46)	4.44 (4.42–4.47)	0.03
Hemoglobin, g/dl^1^	13.6 (13.5–13.7)	13.6 (13.5–13.7)	13.7 (13.6–13.8)	13.6 (13.5–13.8)	0.50
Men^2^					
Serum albumin, g/dl	4.38 (4.35–4.42)	4.45 (4.42–4.49)	4.44 (4.40–4.47)	4.45 (4.41–4.48)	0.02
Hemoglobin, g/dl	14.4 (14.2–14.5)	14.4 (14.2–14.6)	14.4 (14.2–14.6)	14.4 (14.3–14.6)	0.55
Women^2^					
Serum albumin, g/dl	4.42 (4.39–4.45)	4.43 (4.40–4.46)	4.44 (4.41–4.47)	4.44 (4.41–4.47)	0.36
Hemoglobin, g/dl	13.0 (12.9–13.2)	13.0 (12.8–13.1)	13.2 (13.1–13.3)	13.0 (12.9–13.1)	0.57

^1^Age, sex, smoking status, and the use of antidiabetic medications were adjusted

^2^Age, smoking status, and the use of antidiabetic medications were adjusted

Adjusted mean serum albumin levels tended to be lower in individuals with the fewest teeth than in the rest of the participants, although the mean serum albumin levels were within normal limits, and the difference was rather small. This association was significant in men but not in women. In contrast, adjusted mean hemoglobin levels did not differ by the number of teeth.

### Associations according to SES

We employed SES stratification for the intakes of grain products, vegetables, and meat as well as serum albumin levels (Table 5) as these variables showed significant associations with the number of teeth in the preceding analyses. The positive association between the number of teeth and serum albumin levels (trend *p* value: *p* = 0.06 in individuals with a low income and *p* = 0.36 in individuals with a high income)/meat intake (trend *p* value: *p* < 0.01 in individuals with a low income and *p* = 0.48 in individuals with a high income) tended to be more evident in individuals with a low income (interaction *p* value: *p* = 0.07, 0.08, respectively). The positive association between the number of teeth and meat intake (trend *p* value: *p* < 0.01 in individuals with a low household expenditure and *p* = 0.50 in individuals with a high household expenditure) tended to more evident in participants with a low household expenditure (interaction *p* value: *p* = 0.09). Meat intake was the highest among Q4 in participants with high educational attainment (interaction *p* value: *p* = 0.02), although the trend analysis showed an insignificant association between the number of teeth and meat intake for both low and high educational attainment (trend *p* value: *p* = 0.09 and *p* = 0.05, respectively).

**Table 5:** Adjusted means of selected factors according to socioeconomic status (brackets contain 95% confidence intervals)

	Number of teeth				Trend
	Q1	Q2	Q3	Q4	*p* value
Household income^1,2,3^					
Low Serum albumin, g/dl	4.37 (4.33–4.41)	4.44 (4.39–4.49)	4.38 (4.33–4.43)	4.46 (4.40–4.51)	0.06
Grain products, g	467 (442–492)	462 (434–490)	420 (391–449)	420 (388–452)	< 0.01
Vegetables, g	280 (249–312)	310 (275–345)	339 (302–375)	333 (293–372)	0.02
Meats, g	42 (33–51)	56 (46–66)	62 (52–72)	61 (49–72)	< 0.01
High Serum albumin, g/dl	4.42 (4.39–4.45)	4.44 (4.42–4.47)	4.46 (4.43–4.49)	4.44 (4.41–4.46)	0.36
Grain products, g	451 (434–468)	445 (429–461)	432 (417–447)	434 (419–448)	0.08
Vegetables, g	317 (297–338)	336 (316–355)	334 (316–352)	330 (312–347)	0.50
Meats, g	68 (61–75)	66 (60–72)	70 (64–76)	70 (64–76)	0.48
Equivalent household expenditure^4,5,6^					
Low Serum albumin, g/dl	4.40 (4.37–4.43)	4.46 (4.43–4.50)	4.42 (4.39–4.46)	4.44 (4.40–4.47)	0.22
Grain products, g	469 (450–488)	467 (447–488)	454 (432–475)	455 (435–476)	0.23
Vegetables, g	300 (278–323)	329 (305–353)	340 (315–366)	313 (288–337)	0.34
Meats, g	52 (46–59)	61 (54–69)	64 (57–72)	65 (58–72)	< 0.01
High Serum albumin, g/dl	4.40 (4.36–4.44)	4.41 (4.38–4.45)	4.45 (4.42–4.48)	4.45 (4.42–4.49)	0.02
Grain products, g	444 (423–465)	423 (405–442)	413 (397–429)	409 (393–426)	0.01
Vegetables, g	308 (281–335)	331 (306–355)	345 (324–366)	346 (324–367)	0.03
Meats, g	70 (62–79)	61 (54–69)	68 (61–75)	71 (64–77)	0.50
Educational attainment^5,7,8^					
Low Serum albumin, g/dl	4.40 (4.37–4.43)	4.44 (4.41–4.46)	4.42 (4.40–4.45)	4.43 (4.41–4.46)	0.14
Grain products, g	458 (443–472)	454 (439–469)	429 (414–443)	437 (422–452)	< 0.01
Vegetables, g	298 (280–316)	330 (312–348)	335 (317–353)	321 (303–340)	0.06
Meats, g	57 (51–62)	60 (54–66)	67 (61–72)	62 (56–67)	0.09
High Serum albumin, g/dl	4.39 (4.34–4.44)	4.46 (4.41–4.50)	4.48 (4.43–4.52)	4.48 (4.44–4.52)	0.01
Grain products, g	467 (433–501)	414 (386–443)	424 (397–450)	415 (392–438)	0.06
Vegetables, g	321 (279–362)	324 (289–360)	354 (321–386)	357 (328–385)	0.08
Meats, g	70 (56–84)	71 (59–83)	65 (54–76)	86 (77–96)	0.05

^1^Interaction between the number of teeth and household income: serum albumin, *p* = 0.07; grain products, *p* = 0.29; vegetables, *p* = 0.38; meats, *p* = 0.08

^2^Age, sex, smoking status, the use of antidiabetic medications, and the square root of the number of household members were adjusted

^3^Low, < 2 million JPY; high, ≥ 2 million JPY

^4^Interaction between the number of teeth and equivalent household expenditure: serum albumin, *p* = 0.13; grain products, *p* = 0.90; vegetables, *p* = 0.52; meats, *p* = 0.09

^5^Age, sex, smoking status, and the use of antidiabetic medications were adjusted

^6^Low, < 133 thousand JPY; high, ≥ 133 thousand JPY

^7^Interaction between the number of teeth and educational attainment: serum albumin, *p* = 0.59; grain products, *p* = 0.30; vegetables, *p* = 0.45; meats, *p* = 0.02

^8^Low, up to high school; high, college or more

## Discussion

This nationwide cross-sectional study revealed that having fewer teeth was associated with a poorer nutritional status: a lower intake of vegetables and meats as well as animal protein, vitamins, minerals, and dietary fiber. Moreover, it was associated with lower mean blood albumin levels. These associations tended to be more evident in individuals with a low SES.

The associations between oral health, dietary intake, and nutritional status observed in this study are mostly consistent with findings from previous studies in Western countries [3–5, 7–9, 13–17, 20] and Japan [6, 10–12], although there are some methodological differences in study design (cross-sectional vs. longitudinal), assessment of oral health status (self-reported questionnaire, interview, or clinical examination), and dietary assessment (food frequency questionnaire, dietary record, or dietary recall).

Hildebrandt et al. showed that a reduced number of functional units tended to be associated with chewing difficulty, as shown by an avoidance of stringy foods (including meat), crunchy foods (including vegetables), and dry solid foods (including bread) in older people [32]. Other quantitative studies reported that poor oral health was associated with a low intake of certain food groups (fruits, vegetables, meat, beans, and oil) [3–12], a poor nutrient intake (protein, vitamins, carotenoids, dietary fiber, and calcium) [3–7, 9, 10, 12–15], and low blood concentrations of albumin, carotenoids, α-tocophenol, hydroxyvitamin D, vitamin B_6_, vitamin B_12_, folate, and ascorbic acid [4, 16, 17, 20].

Poor oral health was also shown to be associated with a high intake of grain products (mainly rice) in a previous Japanese study [10] and our study, whereas bread was considered a “hard-to-chew food” in a previous study from the USA [32]. The intake of dietary fats was shown to have an inverse association with the number of teeth [3, 7, 9, 13]; however, this association was not observed in our study. Moreover, both inverse [3, 9, 10] and null [5] associations between total energy intake and the number of teeth have been reported, and a significant association was not observed in our study.

The association between having fewer teeth and a lower protein (specifically, animal protein) intake as well as lower serum albumin levels is a notable finding, because a low serum albumin level may be a modifiable risk factor for frailty [18], sarcopenia [33], and mortality [19] in older people. Stratified analysis by sex showed that the significant association between having fewer teeth and low serum albumin levels was observed only in men. Although we assume that the difference in serum albumin levels between Q1 and others among women may be too small to detect a significant difference, it should be further investigated in future studies. The present study included few persons with hypoalbuminemia, likely because the participants were community-dwelling independent adults, including middle-aged individuals; however, lower serum albumin levels, associated with having fewer teeth, should be further investigated to prevent frailty and sarcopenia at an older age.

The association between having fewer teeth and poor nutritional status was particularly evident among individuals with a low SES, which raises an important public health issue. In Japan, dental prostheses are covered by the universal healthcare insurance; however, a social gradient in dental prosthesis use in older adults has been observed in a previous study [34]. We could not obtain information on use of dentures, bridges, or implants in this study; however, it is possible that promoting adequate dental management may improve diet quality in individuals with a low SES and few remaining teeth.

This study has some limitations. Because of its cross-sectional design, we cannot infer a causal relationship between the number of teeth and dietary intake. As we employed a single administration of 1-day dietary records, we could not account for day-to-day variations in dietary intake. Moreover, the use of self-reported tooth counts may not reflect the actual number of teeth. However, previous studies reported a reasonable validity for self-reported tooth counts when compared to tooth counts attained by clinical examination in Japanese adults aged 40–56 years [35] and older adults aged 65 or over [36]. Yamamoto et al. reported that Pearson’s correlation coefficients between self-reported number of teeth and actual number of teeth were 0.81 among older adults [36]. Because we added the definition of “natural teeth” in the questionnaire, and we used quartiles of the number of teeth in the statistical analyses, we believed that misclassification of the rank of the number of teeth would exist but would be acceptable. Finally, we could not adjust for the use of dentures, bridges, or implants in the statistical model because of a lack of information on these variables. Wearing dentures or receiving treatment such as bridges or implants is expected to improve masticatory performance and dietary intake. Generally, people with less teeth are more likely to use dentures, bridges, or implants and more likely to improve masticatory performance and dietary intake. Therefore, adjusting the use of dentures, bridges, or implants would result in larger difference in dietary intake between people with less and more teeth. On the other hand, people with high SES are more likely to use dentures, bridges, or implants and more likely to improve masticatory performance and dietary intake. Therefore, adjusting the use of dentures, bridges, or implants would result in smaller difference in dietary intake between people with high and low SES. In other words, the use of dentures, bridges, or implants would be possible mediating factors in the association between the number of teeth and dietary intake.

This study also has some strengths, including the use of a nationally representative dataset of middle-aged as well as older men and women; a considerable sample size; models that were controlled for age, total energy intake, smoking, and diabetes status; and quantitative dietary assessment using weighed dietary records. It has been pointed that various oral-health factors such as tooth loss, chewing ability, oral pain, xerostomia, and altered taste are associated with dietary intake [36, 37]. When collecting information by self-administered questionnaire survey, tooth loss seems to be more reliable than other factors, because it is more objective than other factors, and several studies evaluated its validity [35, 36] in spite of the limitation caused by self-report as mentioned above.

## Conclusions

Having few remaining teeth was associated with a low nutrient intake and low serum albumin levels. Our findings highlight the importance of promoting oral health in middle-aged and older people to help them maintain adequate nutritional status. Because the association between the number of teeth and dietary intake tended to be more evident in individuals with a low SES, SES should be considered when promoting oral health and dietary strategies.

## Additional file

Additional file 1: **Table S1.** Adjusted means of food group intake by the number of teeth (brackets contain 95% confidence intervals). **Table S2.** Adjusted means of nutrient intake by the number of teeth (brackets contain 95% confidence intervals). (DOCX 47 kb)

## Abbreviations

JPY: Japanese yen
MUFA: Mono-unsaturated fatty acids
NIPPON DATA2010: National Integrated Project for Prospective Observation of Non-communicable Disease and its Trends in the Aged 2010
PUFA: Poly-unsaturated fatty acids
SES: Socioeconomic status
SFA: Saturated fatty acids
