# Supplementary material for: Having few remaining teeth is associated with a low nutrient intake and low serum albumin levels in middle-aged and older Japanese individuals: findings from the NIPPON DATA2010
Source: Environ Health Prev Med. 2019 Jan 5;24:1. doi: 10.1186/s12199-018-0752-x (PMC6320628; doi:10.1186/s12199-018-0752-x)
Supplement: Supplementary file 1 — Table S1. Adjusted means of food group intake by the number of teeth (brackets contain 95% confidence intervals). Table S2. Adjusted means of nutrient intake by the number of teeth (brackets contain 95% confidence intervals). (DOCX 47 kb) [file 12199_2018_752_MOESM1_ESM.docx]

Table S1. Adjusted means of food group intake by the number of teeth (brackets contain 95% confidence intervals).

|  | Number of teeth | | | | | | | | Trend |
| --- | --- | --- | --- | --- | --- | --- | --- | --- | --- |
|  | Q1 | | Q2 | | Q3 | | Q4 | | p-value |
| Grain products, g/1000kcal | 246 | (239-252) | 236 | (229-242) | 223 | (217-229) | 228 | (222-234) | <0.01 |
| Rice, g/1000kcal | 183 | (176-190) | 177 | (170-185) | 163 | (156-170) | 171 | (165-178) | <0.01 |
| Bread, g/1000kcal | 21 | (19-23) | 20 | (18-22) | 20 | (18-23) | 19 | (17-21) | 0.20 |
| Noodles, g/1000kcal | 33 | (28-38) | 32 | (27-37) | 31 | (27-36) | 29 | (24-33) | 0.19 |
| Potatoes, g/1000kcal | 33 | (29-36) | 34 | (31-37) | 31 | (28-34) | 33 | (30-36) | 0.94 |
| Soy and soy products, g/1000kcal | 39 | (35-43) | 36 | (33-40) | 39 | (36-43) | 38 | (34-41) | 0.89 |
| Vegetables, g/1000kcal | 161 | (153-170) | 177 | (169-185) | 178 | (170-186) | 177 | (169-184) | 0.01 |
| Fruits, g/1000kcal | 72 | (66-78) | 77 | (70-83) | 78 | (72-84) | 77 | (71-83) | 0.28 |
| Fishes and Shellfishes, g/1000kcal | 47 | (43-51) | 48 | (44-51) | 50 | (47-54) | 48 | (45-51) | 0.50 |
| Meats, g/1000kcal | 32 | (29-34) | 32 | (30-35) | 33 | (31-36) | 35 | (33-37) | 0.07 |
| Eggs, g/1000kcal | 18 | (16-20) | 20 | (19-22) | 20 | (18-22) | 18 | (17-20) | 0.81 |
| Milks, g/1000kcal | 49 | (43-55) | 59 | (53-65) | 61 | (55-66) | 59 | (53-64) | 0.03 |
| Beverages, g/1000kcal | 376 | (353-400) | 385 | (361-409) | 394 | (371-417) | 403 | (381-426) | 0.09 |
| Age, sex, smoking status and the use of antidiabetic medications were adjusted. | | | | | | | | | |

Table S2 Adjusted means of nutrient intake by the number of teeth (brackets contain 95% confidence intervals).

|  | Number of teeth | | | | | | | | Trend |
| --- | --- | --- | --- | --- | --- | --- | --- | --- | --- |
|  | Q1 | | Q2 | | Q3 | | Q4 | | p-value |
| Carbohydrate, %Energy | 58.6 | (57.8-59.4) | 57.8 | (57.1-58.6) | 56.4 | (55.7-57.1) | 57.2 | (56.5-57.9) | <0.01 |
| Dietary fiber, g/1000kcal | 8.7 | (8.4-9.0) | 9.1 | (8.8-9.4) | 9.1 | (8.9-9.4) | 9.2 | (8.9-9.5) | 0.01 |
| Protein, %Energy | 14.8 | (14.5-15.0) | 15.0 | (14.7-15.2) | 15.3 | (15.1-15.6) | 15.2 | (15.0-15.5) | <0.01 |
| Animal protein, %Energy | 7.3 | (7.0-7.6) | 7.6 | (7.4-7.9) | 8.1 | (7.8-8.4) | 8.0 | (7.7-8.2) | <0.01 |
| Vegetable protein, %Energy | 7.5 | (7.3-7.6) | 7.3 | (7.2-7.5) | 7.2 | (7.1-7.4) | 7.3 | (7.2-7.4) | 0.08 |
| Fat, %Energy | 23.3 | (22.7-23.9) | 23.9 | (23.3-24.5) | 24.3 | (23.8-24.9) | 23.9 | (23.3-24.4) | 0.10 |
| SFA, %Energy | 6.1 | (5.9-6.3) | 6.3 | (6.1-6.5) | 6.4 | (6.2-6.5) | 6.2 | (6.0-6.4) | 0.29 |
| MUFA, %Energy | 7.6 | (7.4-7.9) | 7.9 | (7.7-8.2) | 8.0 | (7.8-8.3) | 8.0 | (7.7-8.2) | 0.04 |
| PUFA, %Energy | 5.5 | (5.3-5.7) | 5.6 | (5.4-5.7) | 5.5 | (5.3-5.7) | 5.5 | (5.4-5.7) | 0.99 |
| Sodium, mg/1000kcal | 2344 | (2273-2415) | 2380 | (2309-2451) | 2387 | (2319-2455) | 2334 | (2266-2401) | 0.85 |
| Potassium, mg/1000kcal | 1304 | (1269-1339) | 1383 | (1348-1418) | 1395 | (1362-1429) | 1396 | (1363-1429) | <0.01 |
| Calcium, mg/1000kcal | 298 | (286-309) | 309 | (297-321) | 319 | (308-330) | 314 | (302-325) | 0.03 |
| Magnesium, mg/1000kcal | 141 | (137-144) | 146 | (142-149) | 147 | (144-151) | 147 | (144-151) | <0.01 |
| Iron, mg/1000kcal | 4.5 | (4.4-4.7) | 4.6 | (4.5-4.8) | 4.7 | (4.6-4.8) | 4.7 | (4.6-4.8) | 0.08 |
| Zinc, mg/1000kcal | 4.2 | (4.1-4.3) | 4.2 | (4.2-4.3) | 4.3 | (4.2-4.4) | 4.3 | (4.3-4.4) | <0.01 |
| Vitamin A, µgRE/1000kcal | 277 | (251-304) | 304 | (278-331) | 331 | (306-356) | 332 | (307-357) | <0.01 |
| β-carotene, µg/1000kcal | 2300 | (2123-2477) | 2539 | (2363-2715) | 2669 | (2500-2837) | 2701 | (2534-2869) | <0.01 |
| Vitamin D, µg/1000kcal | 4.8 | (4.3-5.2) | 4.8 | (4.4-5.3) | 5.1 | (4.6-5.5) | 4.9 | (4.5-5.3) | 0.53 |
| Vitamin E, mg/1000kcal | 4.7 | (3.6-5.7) | 4.6 | (3.5-5.6) | 6.0 | (5.0-7.0) | 6.0 | (5.0-7.0) | 0.02 |
| Vitamin K, µg/1000kcal | 143 | (134-153) | 147 | (137-156) | 150 | (141-159) | 151 | (142-160) | 0.21 |
| Vitamin B_1_, mg/1000kcal | 0.91 | (0.46-1.36) | 1.08 | (0.63-1.53) | 1.26 | (0.83-1.69) | 1.53 | (1.11-1.96) | 0.04 |
| Vitamin B_2_, mg/1000kcal | 0.83 | (0.58-1.08) | 0.86 | (0.61-1.10) | 0.98 | (0.74-1.21) | 1.11 | (0.87-1.34) | 0.08 |
| Vitamin B_6_, mg/1000kcal | 0.90 | (0.45-1.34) | 1.25 | (0.81-1.69) | 1.50 | (1.08-1.93) | 1.80 | (1.38-2.22) | <0.01 |
| Vitamin B_12_, µg/1000kcal | 3.6 | (3.3-3.9) | 3.7 | (3.4-4.0) | 3.8 | (3.5-4.0) | 3.9 | (3.6-4.2) | 0.11 |
| Folic acid, µg/1000kcal | 174 | (167-181) | 188 | (181-195) | 187 | (180-194) | 192 | (186-199) | <0.01 |
| Vitamin C, mg/1000kcal | 71 | (64-79) | 72 | (64-79) | 80 | (72-87) | 78 | (71-85) | 0.11 |
| Abbreviations; SFA, Saturated fatty acids; MUFA, Mono-unsaturated fatty acids; PUFA, Poly-unsaturated fatty acids. | | | | | | | | | |
| Age, sex, smoking status and the use of antidiabetic medications were adjusted. | | | | | | | | | |
